# *In Vitro* Exposure Assessment Improves Hazard Ranking of Bisphenol A Alternatives

**DOI:** 10.1021/acs.chemrestox.6c00300

**Published:** 2026-09-30

**Authors:** Luise Henneberger, Sophia Bardehly, Arina Kremser, Vanessa Srebny, Beate I. Escher

**Affiliations:** † Department of Cell Toxicology, Helmholtz Centre for Environmental Research − UFZ, Permoserstr. 15, 04318 Leipzig, Germany; ‡ Environmental Toxicology, Department of Geosciences, Eberhard Karls University Tübingen, 72076 Tübingen, Germany; § German Center for Child and Adolescent Health (DZKJ), Partner Site Leipzig/Dresden, 04103 Leipzig, Germany

## Abstract

*In vitro* toxicity data can be used for comparative hazard assessment of the problematic plastic chemical bisphenol A (BPA) and its replacement chemicals. Hazard assessment is exclusively based on nominal effect concentrations of the BPA alternatives, but due to their diversity of physicochemical properties and stability, this may lead to inaccurate hazard ranking. In the present study, chemical stability as well as freely dissolved concentrations (*C*_free_) were determined for BPA and 24 of its alternatives. BPA and 22 alternatives were found to be stable in bioassay medium for up to 48 h. The diglycidyl ether of BPA (BADGE) was completely degraded in medium. For BPPH, a loss of up to 37% was observed. A mass balance model was used to derive *C*_free_ of the stable chemicals in the bioassay medium. For model validation, *C*_free_ in bioassay medium, as well as binding to proteins and membrane lipids, was measured for BPA and 15 of the alternatives. Experimentally determined *C*_free_ agreed with model predictions within a factor of 10. Relative effect potencies (REPs) of the BPA alternatives compared to BPA were calculated based on nominal (REP_nom_) and freely dissolved effect concentrations (REP_free_) for two representative end points (activation of estrogen receptor α and inhibition of mitochondrial membrane potential). For hydrophilic and moderately hydrophobic BPA alternatives, REP_nom_ and REP_free_ agreed well. REP_free_ was up to 147× higher compared to REP_nom_ for hydrophobic and highly protein-bound alternatives like TCBPA or BPAF. BP-MIBK had the highest estrogenic potential based on REP_nom_, but the highly protein-bound BPAF was identified as most potent based on REP_free_. TCBPA has a lower estrogenic potential than BPA based on REP_nom_ (0.11), but based on REP_free_ (12.8), it is more potent than BPA. The results of the present study highlight the need to correct for different *in vitro* exposure conditions to allow accurate hazard ranking.

## Graphical abstract

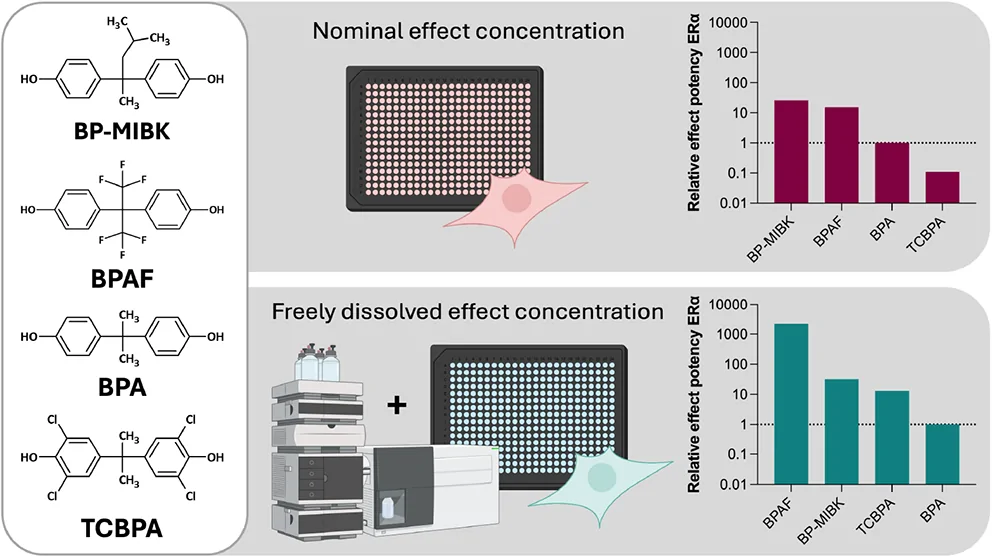

## Introduction

Bisphenol A (BPA) was used for decades as a plastic chemical, but due to its endocrine disrupting properties it is now banned for food-contact materials^1^ and thermal paper^2^ in the European Union. Numerous BPA replacement chemicals have therefore been developed, but for most of them effects to human and environmental health have not been thoroughly assessed.^3,4^ Cell-based *in vitro* bioassays allow a fast screening for adverse effects and can help to identify which alternatives show similar toxicity compared to BPA itself and which ones can be considered more benign. Numerous *in vitro* toxicity studies have been published, but the majority has either focused on only a few of the BPA alternatives or few specific end points like estrogenicity and androgenicity^5,6^ or adipogenesis.^7,8^ The most comprehensive *in vitro* toxicity datasets for BPA and its alternatives can be found in the recent publication by Srebny et al.^9^ that reports data from six assays for BPA and 26 alternatives and in the Tox21 database, which contains data from various assays for BPA and 15 alternatives as reviewed by Adamovsky et al.^4^

To date, comparative hazard assessment using *in vitro* toxicity data for BPA and its alternatives was exclusively done based on nominal effect concentrations.^9–12^ However, nominal concentrations do not necessarily correlate with the bioactive concentration, because the test concentration of the chemicals added at the beginning of the bioassay can be reduced by plastic sorption, volatilization, cellular metabolism, abiotic degradation and binding to cell culture medium constituents (Figure 1).^13^ Some of these binding and loss processes are only relevant for certain compound classes or assay types. For example, plastic sorption was shown to be negligible for assays that use a high serum content, i.e., 10% fetal bovine serum (FBS) in 384-well plates.^14^ Furthermore, many cell lines applied in high-throughput screening assays (e.g., HEK293 cells) have limited metabolic capacity, which makes this loss process also less relevant.^15^ Volatility can be evaluated using the medium-air partition constant (*K*_medium/air_) of the test chemicals. Previous studies have shown that chemicals with log *K*_medium/air_ <4 are lost from the assay plates or even cross-contaminate neighboring wells and cannot be tested in open well plate formats.^16,17^

**Figure 1:**
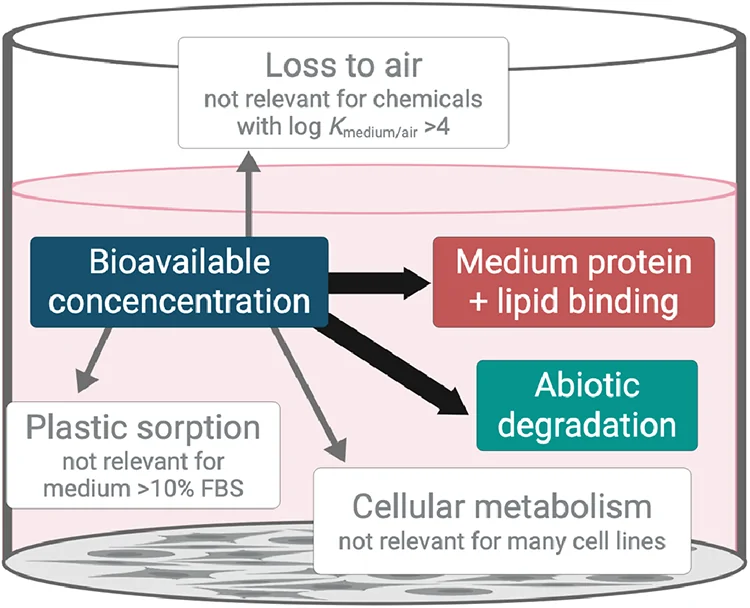
Binding and loss processes in cell-based *in vitro* bioassays and their relevance for BPA and its alternatives. Created in BioRender. Henneberger, L. (2026) https://BioRender.com/squs13m.

Following these considerations, previous studies identified abiotic stability as a key parameter for *in vitro* exposure assessment that should always be measured in the first place.^18–20^ Unfortunately, stability measurements in bioassay medium are rarely published. For BPA and its two alternatives Bisphenol S (BPS) and F (BPF), medium concentrations were recently measured over time by Brenner et al.^21^ and were found to be stable for 24 h. The diglycidyl ether of BPA (BADGE) was reported to hydrolyze quickly in aqueous solution,^9,22,23^ which will occur in medium as well. However, for most BPA replacement chemicals the stability in assay medium is not known.

For stable compounds the binding to medium proteins and lipids will determine the bioavailable concentration. In previous studies, free fractions (*f*_free_) in cell culture medium were measured for BPA, BPS and BPF,^21,24^ but experimental data for other BPA alternatives are lacking. Prediction of *f*_free_ is also possible with a variety of different *in vitro* distribution models,^13^ but the required chemical input parameters for protein and lipid binding are also not available experimentally for the majority of the BPA alternatives, which may lead to high prediction errors.

To address the aforementioned data gaps, BPA and 24 of its alternatives with diverse physicochemical properties were tested for their chemical stability in cell culture medium in the present study. For the subset of stable chemicals, binding to bovine serum albumin (BSA) and phospholipid membranes was quantified. BSA is the most abundant protein in FBS and can be considered to be the predominant sorptive phase for BPA and its alternatives.^25–27^ The measured distribution ratios to proteins and lipids were used to validate the quantitative structure-activity relationships (QSARs) reported in previous studies^28,29^ that allow a prediction of protein and lipid binding from the octanol-water partition constant (*K*_ow_) and speciation of the BPA alternatives. Additionally, *f*_free_ in cell culture medium was measured and compared to *f*_free_ predicted using a simple mass balance model.^30^ The experimental *f*_free_ were used to calculate relative effect potencies (REPs) of the BPA alternatives based on freely dissolved concentrations and the derived REPs were compared to published REPs based on nominal concentrations.

## Materials and Methods

### Chemicals

BPA and 24 alternatives were selected based on the previous work of Srebny et al.^9^ (see also Table 1). Further information on the test chemicals (chemical name and identifiers, purity, provider, Henry constants and p*K*_a_ values) can be found in Table S1. Before starting the experiments, volatility of all chemicals was assessed by calculating log *K*_medium/air_. All test chemicals had log *K*_medium/air_ >4 (Table S1) and can therefore be considered nonvolatile.

**Table 1:** Test Chemicals, Logarithmic Octanol-water (log *K*_ow_) Partition Constants of the Neutral Species, Liposome-water (log *D*_lip/w_) and Bovine Serum Albumin-water Distribution Ratios (log *D*_BSA/w_) at pH 7.4^a^

abbreviation	trivial name	log *K*_ow_ [*L*_w_/*L*_o_]^b^	log *D*_lip/w_ (pH 7.4) [*L*_w_/*L*_lip_]^c^	log *D*_BSA/w_ (pH 7.4) [*L*_w_/*L*_BSA_]^c^
TCBPA	Tetrachlorobisphenol A	6.36	6.10	5.49
TBBPA	Tetrabromobisphenol A	5.91	5.24	4.93
TMBPF	Tetramethyl Bisphenol F	4.37	4.53	3.67
BPPH	Bisphenol PH	6.67	6.85	5.29
BPG	Bisphenol G	6.01	6.19	4.82
BADGE	Bisphenol A diglycidyl ether	3.62	3.78	3.13
BPP	Bisphenol P	6.09	6.27	4.88
Pergafast	Pergafast 201	4.11	3.27	3.47
BTUM	4,4′-Bis(p-tolylsulfonylureido)diphenylmethane	4.82	3.99	4.00
BPT	4,4′-Thiodiphenol	3.29	3.42	2.96
BPF	Bisphenol F	2.71	2.85	2.48
BPZ	Bisphenol Z	4.64	4.80	3.85
BzPB	Benzylparaben	3.30	3.45	2.91
Bz	Benzeneacetic acid	2.06	2.07	2.26
BPAP	Bisphenol AP	5.06	5.22	4.15
BPAF	Bisphenol AF	4.72	4.87	3.90
BP-MIBK	Bisphenol-MIBK	4.47	4.63	3.73
BPB	Bisphenol B	3.72	3.87	3.19
BPA	Bisphenol A	4.25	3.65	3.01
BPE	Bisphenol E	4.05	4.20	3.43
BPS-MPE	Bisphenol S benzyl ether	4.28	4.30	3.91
BPS-MAE	Bisphenol S allyl ether	2.85	2.91	2.79
D8	Bisphenol S isopropyl ether	2.87	2.83	2.88
BPS	Bisphenol S	1.97	1.77	2.14
24BPS	2,4-Bisphenol S	1.90	1.72	2.09

a Chemical identifiers and additional physicochemical properties are in the SI Table S1.^9^

b Data measured or collected by Srebny et al.^9^

c Data from Srebny et al.,^9^ calculated from log *K*_ow_.

Stock solutions of all test chemicals were prepared in methanol (Honeywell), except for Pergafast 201 (Pergafast) and 4,4′-Bis­(p-tolylsulfonylureido)­diphenylmethane (BTUM), which were dissolved in acetonitrile (Honeywell). The stock concentrations ranged from 0.05 g/L to 10 g/L. Sodium chloride (NaCl, Carl Roth) was used for the stability measurements. Bovine serum albumin (BSA) was purchased from Sigma-Aldrich (catalog no. A7030). Solvents and chemicals used for instrumental analysis included LC-MS grade acetonitrile (Honeywell), methanol (Chemsolute), isopropanol (Chemsolute), formic acid (Sigma-Aldrich) and ammonium fluoride (Merck). Purified water was prepared using a Milli-Q water purification system from Merck. The media used for stability measurements and determination of freely dissolved concentrations consisted of (a) 98% Opti-MEM supplemented with 2% charcoal-stripped fetal bovine serum (cs-FBS) and 100 U/mL penicillin–streptomycin (Gibco) and (b) 90% DMEM with GlutaMAX, 10% untreated FBS and and 100 U/mL penicillin–streptomycin (Gibco). These media were chosen, because they are used in two bioassays targeting the activation of the estrogen receptor α (ERα) and disruption of mitochondrial membrane potential (MMP). Both were found to be relevant end points for BPA and/or its replacements.^9^

### Instrumental Analysis

Chemical concentrations in the PBS samples after SPME extraction, in the corresponding sample extracts, as well as in the samples from the stability and membrane partitioning experiments were determined using a liquid chromatography instrument (1260 Infinity II, Agilent) coupled to either a fluorescence detector (1100/1200 FLD), a diode array detector (DAD) or a triple quadrupole mass spectrometer (Triple Quad 6420, Agilent). Further details on the instrumentation including column types, instrumental conditions and limits of quantification (LOQs) are provided in the SI Tables S2-4. Calibration standards with a concentration range of 1–10.000 ng/mL in desorption solvent or PBS (for the determination of isotherms, pin-water distribution and membrane-water partitioning) and acetonitrile blanks were measured together with the samples. The PBS samples were diluted 1:1 or 1:10 with acetonitrile according to the composition of the calibration standards.

### Stability Measurements in Bioassay Medium

Previous studies^20,24^ including the work of Brenner et al.^21^ on BPA, BPS and BPF confirmed that partitioning of the test chemicals to cells does not cause a reduction of the total medium concentration. Consequently, *f*_free_ measurements were conducted in spiked medium only without cells, significantly lowering the experimental effort. For the medium-stability assessment of the BPA alternatives, assay medium was spiked with the stock solutions individually. The extraction of the chemicals was performed directly after spiking (0 h) and after incubation for 48 h at 37 °C. For the extraction, 50 μL of the spiked medium was saturated with 18 mg NaCl and extracted with 400 μL acetonitrile by vortexing for 30 seconds. Samples were centrifuged for 10 min at 4000 rpm, and the acetonitrile supernatant was collected and analyzed by LC–MS. Further details on the analytical methods are provided in the previous section and Tables S2–S4. Depending on the compound, target concentrations in the acetonitrile extract ranged from 125 to 1250 ng/mL to ensure a sufficient signal-to-noise ratio for quantification. All extractions were performed in triplicates. The experiment was repeated in two independent weeks, resulting in six data points per compound and time point.

### Solid-phase Microextraction Method

Solid-phase microextraction (SPME) was used to determine protein binding and freely dissolved concentrations (*C*_free_) in bioassay medium of BPA and its alternatives. Only chemicals with a log *K*_ow_ between 1.90 and 5.06 were measured due to the applicability range of the SPME method regarding hydrophobicity. Supel BioSPME 96-Pin Devices from Supelco coated with C18/PAN were used. The 96-pin SPME protocol was established in previous studies^18,20^ and adapted to the selected test compounds as described in SI section S1. The determined uptake kinetics and sorption isotherms can be found in Figures S1–S3. In short, the samples were transferred to the sample plate, shaken for 3 min at 1000 rpm and preincubated at 37 °C for 15 min. Prior to extraction, the pin device was conditioned in a glass-coated deep-well plate containing 800 μL of isopropanol per well for 20 min followed by a wash step in a glass-coated deep-well plate containing 900 μL of Milli-Q water per well for 10 sec. The extraction was performed in a sample plate containing 600 μL of the sample aliquot by immersing the pin device directly into the plate. Plates with pin device were incubated at 37 °C and 1000 rpm for a defined extraction time (Table S5) on a Bioshake Q1 orbital shaker (QInstruments). For desorption, the pin device was transferred to a separate desorption plate with 600 μL desorption solution per well (Table S5) and shaken at 10 °C and 1000 rpm for 2h. During the shaking process the pin device was sealed on top of the plates with laboratory sealing film (Parafilm) to prevent evaporation. Concentrations of the chemicals in the SPME extracts were measured as described in the instrumental analysis section.

### Determination of Freely Dissolved Concentrations and Bovine Serum Albumin Binding

For the determination of *C*_free_, samples were prepared by pipetting a defined amount of the methanolic stock solution to a sample vial, evaporating the solvent and adding 2 mL of medium followed by thorough mixing. Test concentrations were selected based on the nominal effect concentrations (IC_10_ and EC_10_) reported by Srebny et al.^9^ Two to five different concentrations were measured for each chemical, which varied by 1–3 orders of magnitude. The sample solutions were transferred in duplicates into a sample plate and extracted with the SPME pin workflow as described in the previous section.

For the BSA binding experiments, the same test concentrations were used as in the medium experiments conducted to determine *C*_free_. Protein solutions were prepared in PBS with a BSA concentration of 1.56 g/L to simulate the protein content in the medium. The protein concentration of the medium was measured using the Pierce BCA Assay Kit from Thermo Scientific. The methanolic stock solution was added to the sample vial and the solvent was evaporated. 2 mL of the BSA solution were added and samples were vortexed and transferred to the sample plate in duplicates for extraction following the SPME pin workflow. For each test chemical all samples for the determination of *C*_free_ and the BSA binding experiments were extracted on the same day and the resulting extracts were considered technical replicates.

### Determination of Membrane-water Partition Constants

The partitioning of BPA and 15 alternatives to phospholipid membranes was determined with the TRANSIL Membrane Affinity Assay (Sovicell GmbH, Leipzig, Germany). The kit consisted of 96 glass vials with 12 individual units of 8 vials with a total volume of 500 μL 10 mM phosphate buffered saline (pH 7.4) per vial allowing for the testing of 12 different compounds. Each test unit contained 6 vials with increasing amount of membrane vesicles reconstituted from egg yolk and stabilized on porous silica beads. Two vials without beads served as references for each chemical. The affinity to phospholipid membranes was evaluated based on the distribution ratio of the chemical between the immobilized membrane and the aqueous buffer. The assay is applicable for compounds with a log *K*_ow_ between 1.5 and 5.5.^31,32^

The protocol was based on the procedure provided by the company but was adapted as described in the following section. First the test units were thawed overnight at 4 °C and washed afterwards to remove any detached phospholipids. To wash the beads the vials were centrifuged for 10 min at 750×g in a swing-out centrifuge (Thermo Scientific) and 300 μL of the supernatant were removed carefully. After 700 μL of fresh PBS was added to the vials, they were centrifuged again and the supernatant was discarded leaving a volume of 200 μL PBS in the vial. 300 μL fresh PBS was added, leading to a final volume of 500 μL, and the vials were centrifuged quickly for 5 sec at 750*g*, to ensure that all liquid was collected at the bottom. A solution of the test chemical in methanol (250 μM) was prepared and 10 μL of the stock was transferred to each vial of the test unit yielding a final test concentration of 5 μM. This concentration was chosen to ensure a reliable quantification. The methanol concentration in the samples was 2%. Two PBS controls and two solvent controls in acetonitrile with the same chemical concentration as the samples were included for quality control. All samples were incubated on a shaker at room temperature at 100 rpm for 12 min. The sample vials were centrifuged for 10 min at 750*g* and 200 μL of the supernatant was transferred to vials with insert for instrumental analysis. Prior to analytical quantification the PBS samples were diluted 1:1 or 1:10 with acetonitrile. All samples were measured as technical replicates on the same day.

### Data Evaluation

#### Stability Measurements in Bioassay Medium

The loss of the test substances over 48 h (%loss_48h_) was calculated based on the measured concentrations at the beginning (*C*_0 h_) and at the end of the incubation period (*C*_48 h_). Measured concentrations were normalized to nominal concentrations prior to calculating the percentage loss. Loss was calculated for individual measurements as well as for the mean values of all six measurements (eq 1).

1 $${\%\;\mathrm{loss}}_{48\;h}=\left(1-\frac{C_{48\;h}}{C_{0\;h}}\right)\times 100$$

The propagated standard deviation of the loss (*σ*_loss_48 h__) was calculated using eq 2 based on the standard deviations of the mean measured concentrations at 0 and 48 h (*σ*_*C̅*_0 h__ and *σ*_*C̅*_48 h__).

2 $$\sigma_{{\mathrm{loss}}_{48\;h}}=\sqrt{{\left(\frac{{\mathrm{C̅}}_{48\;h}}{{{\mathrm{C̅}}_{0\;h}}^{2}}\times \sigma_{{\mathrm{C̅}}_{0\;h}}\right)}^{2}+{(\frac{1}{{\mathrm{C̅}}_{0\;h}}\times \sigma_{{\mathrm{C̅}}_{48\;h}})}^{2}}$$

#### Determination of Freely Dissolved Concentrations and Bovine Serum Albumin Binding

The determined concentration in the pin coating (*C*_pin_, for details see SI section S1) was used to calculate *C*_free_. Following the approach described by Henneberger et al.,^20^ four different equations were applied depending on the extraction behaviour of the chemical. First the fraction of the chemical in the pin coating was calculated based on the mass balance (eq 3). The total amount of chemical in the sample (*n*_total_) was calculated from the nominal concentration (*C*_nom_), because all chemicals tested with SPME were shown to be stable. *n*_pin_ is the amount of chemical in the pin coating and *V*_sample_ is the volume of the medium sample used for SPME.

3 $$f_{\mathrm{pin}}=\frac{n_{\mathrm{pin}}}{n_{\mathrm{total}}}=\frac{C_{\mathrm{pin}}\times V_{\mathrm{pin}}}{C_{\mathrm{nom}}\times V_{\mathrm{sample}}}$$

The extraction was considered non-depletive if *f*_pin_ < 5%. For calculation of *C*_free_ the chemicals were grouped according to their extraction profile (see also Table S5): (I) non-depletive, linear (eq 4), (II) depletive, linear (eq 5), (III) non-depletive, nonlinear (eq 6), and (IV) depletive, nonlinear (eq 7). The freely dissolved fraction *f*_free_ in medium was calculated by dividing *C*_free_ by *C*_nom_.

$$C_{\mathrm{free}}=\frac{C_{\mathrm{pin}}}{D_{\mathrm{pin}/w}}$$ 4

5 $$C_{\mathrm{free}}=\frac{n_{\mathrm{total}}}{D_{\mathrm{pin}/w}\times \left(\frac{n_{\mathrm{total}}}{C_{\mathrm{pin}}}-V_{\mathrm{pin}}\right)}$$

6 $$C_{\mathrm{free}}={\left(\frac{C_{\mathrm{pin}}}{K_{\mathrm{Fr}}}\right)}^{1/n_{\mathrm{Fr}}}$$

7 $$C_{\mathrm{free}}=\frac{n_{\mathrm{total}}}{C_{\mathrm{pin}}\times {\left(\frac{C_{\mathrm{pin}}}{K_{\mathrm{Fr}}}\right)}^{-1/n_{\mathrm{Fr}}}\times \left(\frac{n_{\mathrm{total}}}{C_{\mathrm{pin}}}-V_{\mathrm{pin}}\right)}$$

SPME was also used to determine the freely dissolved concentration in the presence of BSA. The concentration in the water phase (*C*_w_) of the BSA samples was calculated using the same approach as for *C*_free_ in medium, applying the appropriate equation based on the chemical’s extraction profile regarding depletion and pin sorption linearity. The mass balance was used to calculate the concentration of the test chemical bound to BSA (*C*_BSA_, eq 8). n_w_ is the amount of chemical in the water phase calculated from *C*_w_ and *V*_BSA_ is the total volume of BSA in the sample.

8 $$C_{\mathrm{BSA}}=\frac{n_{\mathrm{total}}-n_{\mathrm{pin}}-n_{w}}{V_{\mathrm{BSA}}}$$

The concentration dependence of the BSA-water distribution ratio (*D*_BSA/w_) was fitted using the log-transformed Freundlich model (eq 9). Binding to BSA was considered linear or nonlinear based on the criteria described for the pin sorption isotherms in SI section S1.

9 $$\log C_{\mathrm{BSA}}=n_{\mathrm{FR}}\times {\log C}_{w}+\log K_{\mathrm{Fr}}$$

For chemicals with concentration independent BSA binding, the *D*_BSA/w_ was calculated from the chemical concentration bound to BSA and the corresponding measured concentration in the water phase by eq 10.

10 $$D_{\mathrm{BSA}/w}=\frac{C_{\mathrm{BSA}}}{C_{w}}$$

#### Determination of Membrane-water Partition Constants

The chemical concentration bound to the phospholipid membrane (*C*_m_) was calculated using the mass balance (eq 11). *V*_m_ represents the total volume of phospholipids on the beads, as provided by the company.

11 $$C_{m}=\frac{n_{\mathrm{total}}-n_{w}}{V_{m}}$$

Equation 12 was used to calculate the bound fraction of the chemical for the six different amounts of TRANSIL beads.

12 $$f_{\mathrm{bound}}=\frac{n_{\mathrm{total}}-n_{w}}{n_{\mathrm{total}}}$$

The membrane-water distribution ratio (*D*_m/w_) was derived from *C*_m_ and *C*_w_ by eq 13. Note that, *D*_m/w_ is comparable, but not identical to the liposome-water distribution ratio (*D*_lip/w_) shown in Table 1, because TRANSIL uses membrane vesicles reconstituted from egg yolk and the *D*_lip/w_ reported in Table 1 were predicted values für palmitoyl-oleyl-phosphatidylcholine (POPC) liposomes and only the value of BPA was experimental.^33^

13 $$D_{m/w}=\frac{C_{m}}{C_{w}}$$

#### Mass Balance Model and Relative Effect Potencies (REPs)

A mass balance model was used to calculate *f*_free_ of the chemicals in assay medium using the volume fractions of water, proteins and lipids in the assay medium as well *D*_BSA/w_ and *D*_m/w_ of the chemicals as input parameters (eq 14).

14 $$f_{\mathrm{free}}={\left(1+D_{\mathrm{BSA}/w}\times \frac{V_{\mathrm{protein},\mathrm{medium}}}{V_{w,\mathrm{medium}}}+D_{m/w}\times \frac{V_{\mathrm{lipid},\mathrm{medium}}}{V_{w,\mathrm{medium}}}\right)}^{-1}$$

For comparison of modelled and measured distribution ratios, *f*_free_ and *C*_free_ the absolute average fold error was calculated (AAFE, eq 15).

15 $$\mathrm{AAFE}=10^{\left[\sum \left|\log_{10}\left(\frac{\mathrm{modeled}\;\mathrm{value}}{\mathrm{measured}\;\mathrm{value}}\right)\right|/\mathrm{number of values}\right]}$$

The relative effect potencies of the BPA alternatives were calculated by dividing the determined effect concentration of BPA by the effect concentration of the BPA alternatives (BPX, eq 16). REPs were derived using the nominal (REP_nom_) and freely dissolved effect concentrations (REP_free_).

16 $$\mathrm{REP}=\frac{{\mathrm{EC}}_{\mathrm{BPA}}}{{\mathrm{EC}}_{\mathrm{BPX}}}$$

## Results

### Stability Measurements in Bioassay Medium

BPA and 22 of its alternatives were found to be stable in bioassay medium (OptiMEM base medium supplemented with 2% cs-FBS). The total medium concentration for these chemicals determined after 48 h of incubation at 37°C differed less than 20% from the total medium concentration determined in the freshly prepared medium (Fig. 2). For the most hydrophobic test chemical Bisphenol PH (BPPH) losses of 37% and 10% were observed in assay medium with 2% and 10% FBS, respectively. BADGE was completely degraded within 48 h in both types of media (Fig. 2).

**Figure 2:**
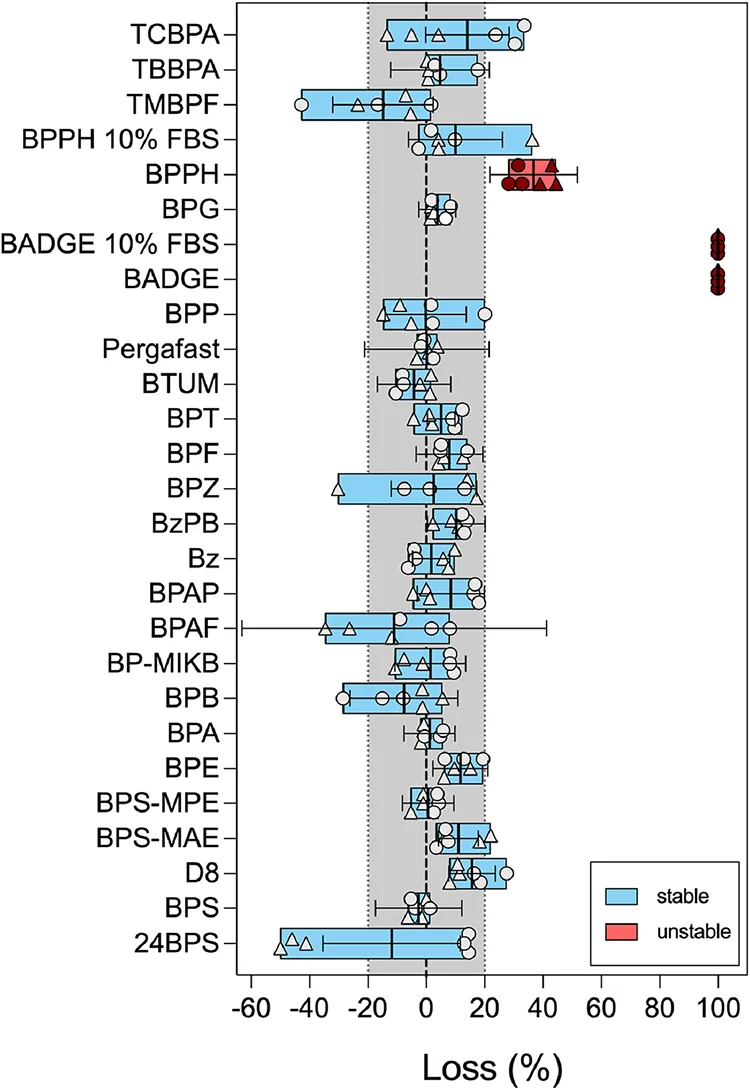
Stability of BPA and 24 alternatives at 37°C in assay medium (OptiMEM supplemented with 2% cs-FBS) expressed as %loss after 48 h, different symbols indicate independent experimental replicates. Stability of BADGE and BPPH was also tested in assay medium consisting of 90% DMEM with 10% untreated FBS.

### Bovine Serum Albumin Binding and Membrane-water Partitioning

The SPME method was successfully optimized for BPA and 15 of the alternatives. Binding to the pin coating as well as equilibration times were found to increase with increasing hydrophobicity (Figure S4). Chemical recoveries (sum of amount in PBS and pin coating) for the optimization experiments in PBS were high (76-111%). Several chemicals showed nonlinear sorption to the pin coating, which had to be considered in the *C*_free_ calculation (Figure S3). Despite the good performance of the SPME method, the testable concentration range was limited for some BPA alternatives due to high LOQs (e.g., 1 μg/mL for BPP and 0.5 μg/mL for BPZ).

Binding to BSA was measured for BPA and 15 of the alternatives (Figure S5, abbreviations in Table 1). For BPF, BPAF, BP-MIBK, BPA, BPS-MAE and D8 binding isotherms were linear, while for BPT, BPZ, BzBP, Bz, BPAP, BPB, BPE and BPS-MPE binding to BSA was concentration-dependent (Table S6). Table 2 reports the mean *D*_BSA/w_. BPS and 24BPS showed weak affinities for BSA. For BPS *D*_BSA/w_ could only be determined at one concentration level and no isotherm was fitted. For 24BPS no binding could be measured at any of the tested concentrations indicating *f*_bound_ < 20% at the tested BSA concentration of 1.56 g/L. Figure 3A shows a comparison of the measured *D*_BSA/w_ with the values predicted from log *K*_ow_ by Srebny et al.^9^ Because regression models based on log *K*_ow_ do not consider concentration dependence, the average *D*_BSA/w_ was calculated and used for the comparison in Fig. 3A even for the chemicals with nonlinear binding isotherms, resulting in generally larger error bars (see also Table 2). On average, the measured *D*_BSA/w_ agreed with the modelled values within a factor of 4.7 (AAFE). For most chemicals the measured and predicted *D*_BSA/w_ agreed within a factor of 7. For benzylparabene (BzBP), BPS-MAE and D8 the measured *D*_BSA/w_ were a factor of 17, 24, and 35 higher compared to the value predicted from log *K*_ow_. For BPA the measured log *D*_BSA/w_ of 3.57 agrees well with literature data^29^ (*D*_BSA/w_ = 3.65). This literature value was also used for the comparison in Figure 3A.

**Figure 3:**
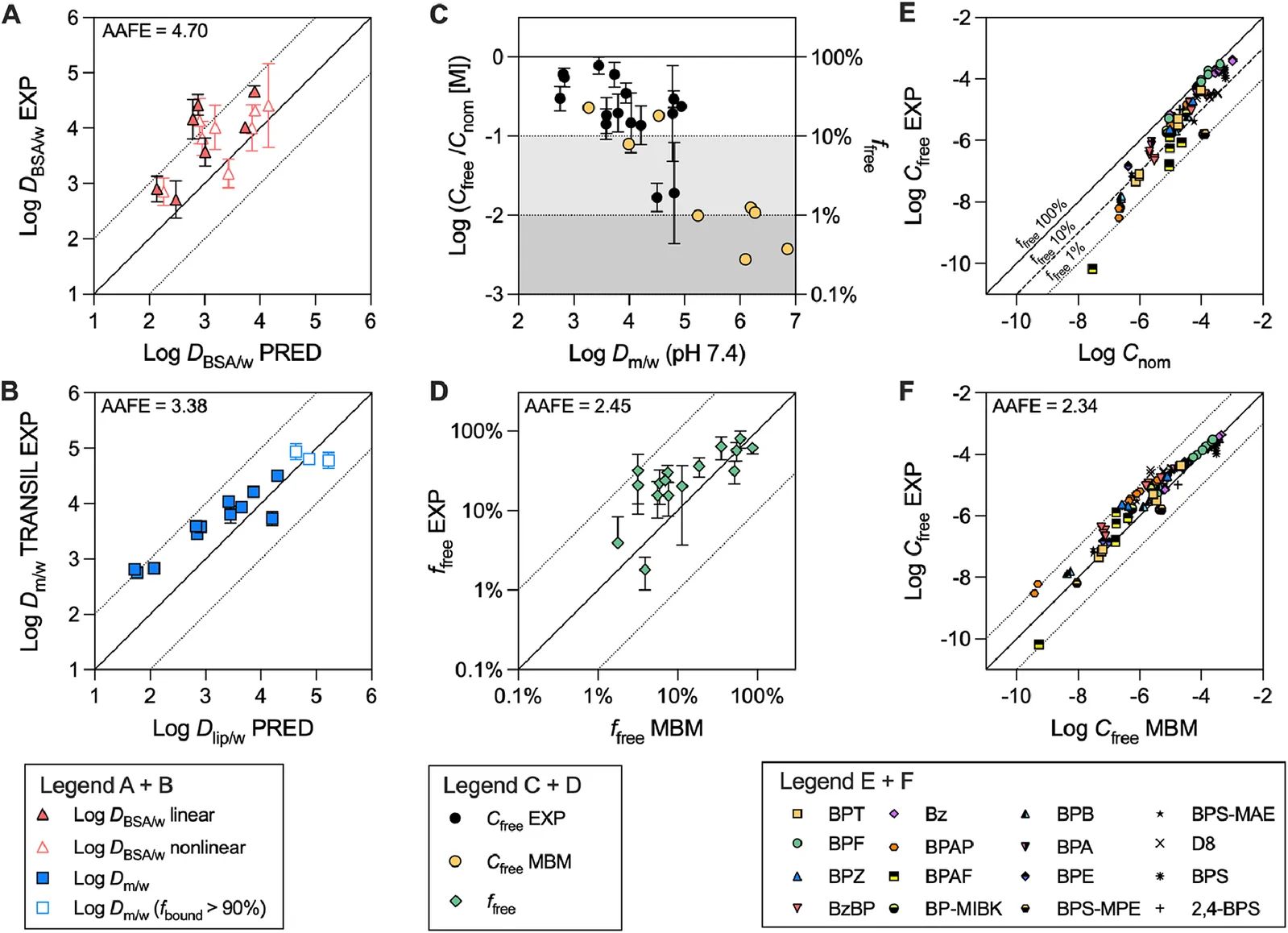
Comparison of measured (EXP) and modelled (MBM) (A) bovine serum albumin-water distribution ratios (*D*_BSA/w_) and (B) membrane or liposome-water distribution ratios (*D*_m/w_ or *D*_lip/w_), (C) dependence of measured (EXP) and modelled (MBM) free fractions (*f*_free_) in assay medium (OptiMEM base medium +2% cs-FBS) of the test chemicals on the membrane-water distribution ratio (*D*_m/w_), (D) comparison of average measured (EXP) and modelled (MBM) *f*_free_, (E) comparison of measured freely dissolved concentrations (*C*_free_) with dosed nominal concentration (*C*_nom_), (F) comparison of measured (EXP) and modelled (MBM) *C*_free_. Solid lines in A, B, D, E and F indicate the 1:1 line, the dotted lines indicate the 1:10 and 10:1 lines. AAFE (absolute average fold error).

**Table 2:** Experimentally Determined Bovine Serum Albumin-water (log *D*_BSA/w_) and Membrane-water Distribution Ratios (log *D*_m/w_) and Measured Free Fractions in Assay Medium (*f*_free_)­^a^,^b^,^c^

chemical	log *D*_BSA/w_ (pH 7.4) [*L*_w_/*L*_BSA_]	SD	N	BSA isotherm	log *D*_m/w_ (pH 7.4) [*L*_w_/*L*_lip_]	SD	N	*f*_free_ medium^c^	SD	N
BPT	3.82	0.31	8	nonlinear	4.03	0.10	8	20%	16%	8
BPF	2.71	0.34	8	linear	3.45	0.04	10	80%	20%	8
BPZ	4.00	0.42	4	nonlinear				30%	7%	4
BzPB	4.13	0.41	10	nonlinear	3.80	0.15	10	22%	10%	10
Bz	2.85	0.25	8	nonlinear	2.83	0.10	8	57%	15%	8
BPAP	4.41	0.76	8	nonlinear	4.78^c^	0.14	4	32%	19%	8
BPAF	4.67	0.10	6	linear	4.81^c^	0.07	4	4%	4%	8
BP-MIBK	4.02	0.05	6	linear	4.94^c^	0.15	4	24%	3%	6
BPB	4.01	0.40	8	nonlinear	4.21	0.08	6	15%	7%	8
BPA	3.57	0.25	8	linear	3.94	0.08	8	36%	10%	8
BPE	3.18	0.26	8	nonlinear	3.73	0.13	10	63%	20%	8
BPS-MPE	4.32	0.10	6	nonlinear	4.50	0.07	4	1.8%	0.8%	6
BPS-MAE	4.16	0.36	10	linear	3.58	0.07	10	16%	7%	10
D8	4.42	0.19	8	linear	3.59	0.08	10	21%	12%	8
BPS	2.90	0.23	6	not measured	2.75	0.04	8	31%	10%	6
24BPS					2.81	0.04	8	61%	9%	8

a SD – standard deviation, N – number of technical replicates.

b *f*_bound_ > 90%.

c Medium: OptiMEM + 2% cs-FBS.

Membrane-water distribution ratios were successfully measured for BPA and 14 of the alternatives using the TRANSIL kit (Tables 2 and S7). For the calculation of *D*_m/w_ only values with *f*_bound_ 20–90% were included. For BPAP, BPAF and BP-MIBK *f*_bound_ was >90% for all tested TRANSIL concentrations and only the two lowest concentrations were used for the calculation. For BPZ no calculation was possible, because the concentrations in the PBS phase were below the detection limit. On average, measured *D*_m/w_ agreed with the values predicted from log *K*_ow_ within a factor of 3.38 (AAFE, Figure 3B). Only for the two hydrophilic alternatives BPS and 24BPS the measured values were a factor of 10 and 12 higher compared to the prediction.

### Freely Dissolved Concentrations in Assay Medium

As shown in Figure 3C, *f*_free_ in assay medium (OptiMEM base medium +2% cs-FBS) decreased with increasing hydrophobicity of the test chemicals. For BPA and 15 alternatives *f*_free_ was measured in the present study with the SPME method. The average measured *f*_free_ in medium ranged from 1.8% (BPS-MPE) to 80% (BPF, see also Table 2, individual values are shown in Table S8). For the remaining eight stable chemicals, including mainly the more hydrophobic alternatives, *f*_free_ was only predicted using eq 14 and the derived values ranged from 0.3% (TCBPA) to 23% (Pergafast). The measured *f*_free_ agreed on average within a factor of 2.45 (AAFE) with the model prediction (Figure 3D). For BPAP and D8, the predicted value was 10 and 6.5-times lower than the measured value. For several chemicals *f*_free_ was found to be dependent on the applied chemical concentration, which agrees with the results of the BSA binding experiments. For chemicals with nonlinear binding isotherms the measured *C*_free_ increased nonlinearly with increasing nominal concentrations (Figure 3E, individual plots are shown in Figure S6). Nonlinear binding was most pronounced for BPAP and BPAF in the measured concentration range. For these two chemicals *f*_free_ ranged from 1.5–54% and 0.2–14% (lowest to highest concentration), respectively. To better account for the concentration dependence of *f*_free_, the model prediction was also done for the individual concentration levels using concentration dependent values for *D*_BSA/w_ as input parameters. Again, there was a good agreement of measured and modelled *C*_free_ (AAFE = 2.34), but BPAP and D8 were still overpredicted by a factor of 10 (Figure 3F).

## Discussion

BPA alternatives represent a diverse group of chemicals. The hydrophobicity of the compounds selected for the present study ranged from log *K*_ow_ 1.9 (24BPS) to 6.7 (BPPH, see also Table 1) and they showed different degrees of ionization at pH 7.4 from >99% neutral (BPP) to >99% negatively charged (Pergafast) or double negatively charged (BTUM, see also Table S1). Consequently, exposure conditions also varied for the different classes of replacement chemicals. As highlighted in two previous studies from our group, exposure and consequently also hazard assessment is most challenging for chemicals that are degraded rapidly during the bioassay.^18,20^ In the present study, complete degradation of BADGE was observed within 48 h. The degradation is most likely the result of hydrolysis at the epoxide rings leading to the formation of different diols.^23^ For BADGE no further analysis apart from stability testing was performed, because exposure assessment can only help to identify problematic chemicals, but cannot solve the general problems that arise for abiotically instable chemicals. Any *in vitro* effects observed for BADGE cannot be linked directly to the applied nominal concentration, because of the rapidly decreasing concentration of BADGE and the increasing concentrations of the transformation products. Depending on the end point of the assay, the measured toxicity might either be caused by the parent chemical BADGE, by its degradation products or by the mixture of both. For example, genotoxic effects reported for BADGE are caused by DNA adducts formed by the parent compound.^34^

For the most hydrophobic BPA alternative of the present study (BPPH) a 37% decrease in medium concentrations was measured after 48 h in medium with 2% FBS. The loss was unlikely caused by abiotic degradation processes, but rather by slow precipitation in medium or binding to the vessel. This is also supported by the fact that a lower loss (only 10%) was observed in medium with 10% FBS. Therefore, it is still reasonable to derive freely dissolved effect concentrations of BPPH based on the mass balance.

The experimental *D*_BSA/w_ and *D*_m/w_ of the BPA alternatives generally agreed well with the values predicted using simple QSARs based on log *K*_ow_ and speciation of the chemical. For future studies, especially when screening large numbers of chemicals, it is therefore feasible to use predicted values for *D*_BSA/w_ and *D*_m/w_. However, it should be noted that the log *K*_ow_ values used for the prediction of *D*_BSA/w_ and *D*_m/w_ were determined experimentally for the majority of the chemicals by Srebny et al.^9^ and some differed quite substantially from predicted log *K*_ow_. Larger uncertainties are expected for chemicals without experimental log *K*_ow_. Further improvement of the prediction may be achieved by using fragment-based QSAR models that take into account the different substitutions of the BPA alternatives. Furthermore, nonlinear protein binding was observed for 8 of the 16 BPA alternatives tested. Concentration-dependent binding cannot be easily predicted from the molecular structure, but is very common for organic acids.^25,35,36^ Results for *D*_m/w_ determined with the TRANSIL kit agreed best with QSAR model predictions for BPA alternatives with moderate hydrophobicity (log *D*_m/w_ 2.5–4.5). Very hydrophilic and hydrophobic chemicals are more challenging to assess experimentally and were outside the applicability domain of the TRANSIL method.

The *D*_BSA/w_ and *D*_m/w_ of the BPA alternatives can in principle be used to predict *f*_free_ or *C*_free_ for any given cell culture medium, provided that the medium protein and lipid content is known. The protein and lipid binding data is also crucial for quantitative *in vitro*-to-*in vivo* extrapolation (QIVIVE) of *in vitro* toxicity data for BPA and its alternatives.^37^ Given that *in vivo* plasma concentrations may be orders of magnitude lower than the *in vitro* effect concentrations, the calculation of *f*_free_ needs to account for the concentration-dependent protein binding. For BPA alternatives that showed nonlinear protein binding *D*_BSA/w_ measured at chemical:BSA ratios (mol_chemical_:mol_BSA_) ν<0.1 should be used for the calculation of *in vivo f*_free_ (see also Table S6). Furthermore, the most hydrophobic BPA alternatives TCBPA, TBBPA, TMBPF, BPPH and BPG have no measured *D*_BSA/w_ and *D*_m/w_. Therefore, experimental validation of *C*_free_ should be conducted in future studies. C18-coated SPME fibers can be applied for hydrophobic chemicals as well,^38^ but the pin device used in the present study is not recommended as slow diffusion into the plastic core material of the pins may hamper equilibration with the SPME device.

As expected, the large differences in hydrophobicity of the BPA alternatives resulted in bioavailable fractions, i.e., *f*_free_, that varied by more than two orders of magnitude from >70% (Bz) to <0.3% (TCBPA). The experimental data for *f*_free_ of BPA determined in the present study (33%) agreed well with the data from a previous study (38%).^24^ For BPF and BPS *f*_free_ of 60 and 37% were determined for medium supplemented with 2% FBS. Brenner et al.^21^ reported *f*_free_ of 43-46% and 46-55% for BPF and BPS for cell culture medium with 10% FBS, which can be considered comparable taking into account that different methods were used for determination of *f*_free_ (SPME vs. equilibrium dialysis) and that the composition of FBS is highly variable.

For comparison of effect data from *in vitro* cell-based bioassays for BPA and its alternatives the relative effect potency (REP) was calculated using eq 16. Using the reported effect concentrations of Srebny et al.^9^ the REPs of the BPA alternatives were calculated for two end points, activation of the estrogen receptor α (ERα) and inhibition of mitochondrial membrane potential (MMP). These two end points were chosen, because BPA and many of the alternatives showed specific activity. For the calculation of REP_free_ for the ERα activation the EC_free_ of BPA and all active alternatives were calculated using two different approaches: (i) using measured *f*_free_ values at the same or similar concentration as the EC (i.e., concentration dependence of *f*_free_ was considered, only available for 16 of the chemicals), and (ii) using *f*_free_ derived with the mass balance model and the average *D*_BSA/w_ (i.e., concentration dependence of *f*_free_ was ignored, eq 14). The second approach was also applied to calculate EC_free_ of BPA and all active alternatives in the MMP assay, because a medium with 10% FBS was used in this assay. The *f*_free_ values used to derive REP_free_ are also depicted in Figure 4A.

**Figure 4:**
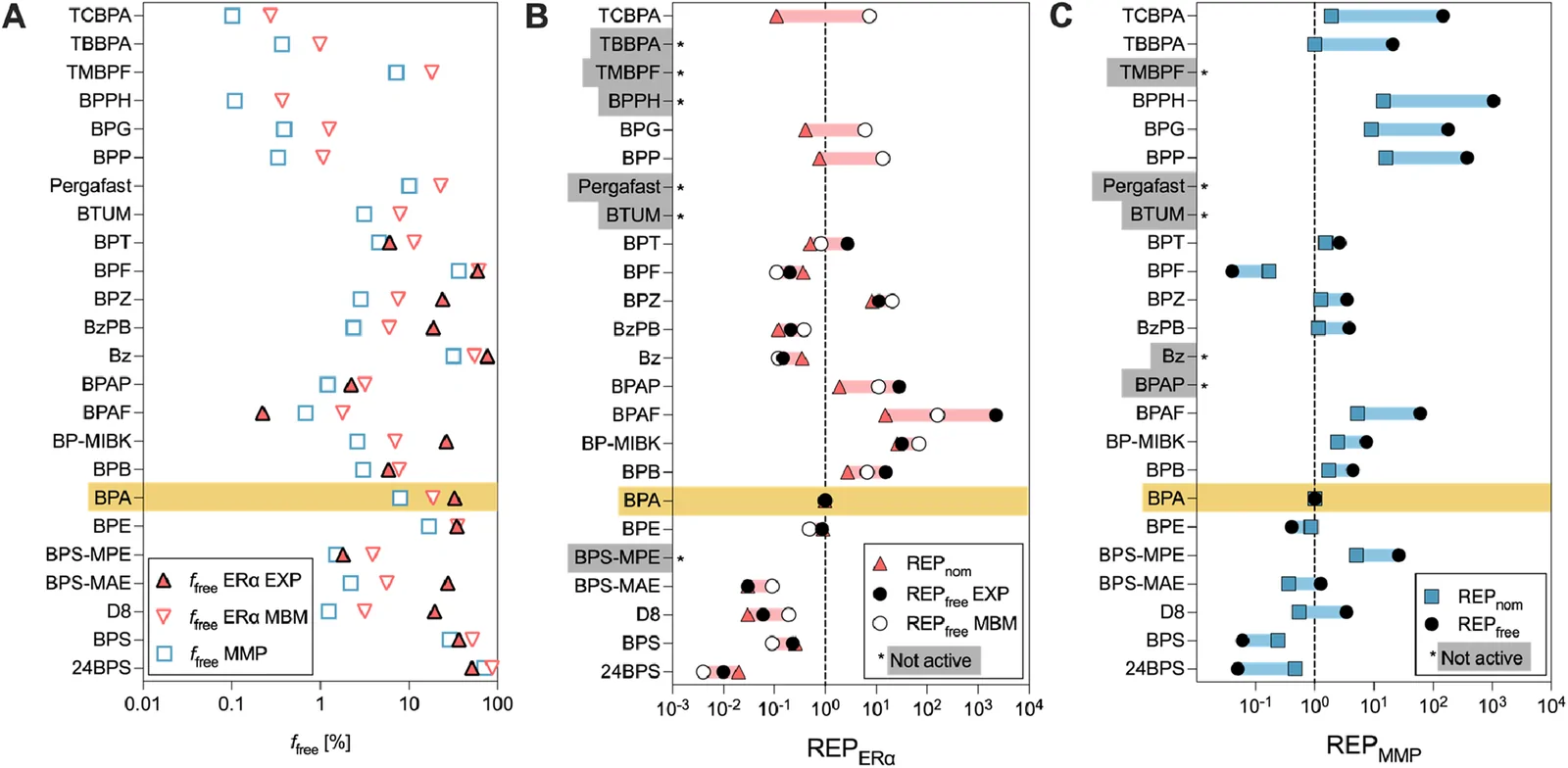
(A) Free fractions (*f*_free_) in assay medium of the ERα (experimentally determined – EXP or calculated by the mass balance model – MBM) and MMP assay (all calculated by MBM) and relative effect potencies (REP) of BPA alternatives that (B) activate ERα *in vitro* (REP_ERα_) or (C) disrupt the mitochondrial membrane potential (REP_MMP_), both calculated based on the nominal (REP_nom_) and freely dissolved effect concentrations (REP_free_).

Figure 4B+C show the derived REPs for both end points. For many BPA alternatives the REP_nom_ and REP_free_ are very similar. This applies mainly to the hydrophilic and moderately hydrophobic BPA alternatives BPF, BPZ, BzBP, Bz, BP-MIBK, BPE, BPS-MAE, D8, BPS and 24BPS that have *f*_free_ similar to BPA itself in both assay media. For BPF, BPS and 24BPS REP_free_ in the MMP assay was slightly lower compared to REP_nom_, because these chemicals have a lower affinity to proteins and consequently a higher *f*_free_ compared to BPA in the MMP assay medium. For the hydrophobic (TCBPA, TBBPA, BPPH, BPG, BPP) and strongly protein-bound BPA alternatives (BPAP, BPAF), the *f*_free_ in both assay media was much lower than the measured *f*_free_ of BPA. Consequently, REP_free_ was much higher than REP_nom_ for the hydrophobic chemicals. For BPAF and TCBPA REP_nom_ and REP_free_ differed by a factor of 147 and 119 for the ERα assay. For the MMP assay REP_nom_ and REP_free_ of the hydrophobic alternatives TCBPA and BPPH differed by a factor of 78 and 73. BP-MIBK, BPAF and BPZ show the highest activation of ERα, if the chemicals are ranked by REP_nom_. Based on REP_free_ the most potent chemicals in ERα were BPAF>BP-MIBK>BPAP (first approach with concentration dependent *f*_free_) and BPAF>BP-MIBK>BPZ (second approach with *f*_free_ predicted by MBM). Interestingly, BPAF is the most potent agonist of ERα, irrespective whether its highly concentration dependent binding to proteins is considered or not in the *f*_free_ calculation. However, the absolute REP increases by a factor of 14, if the concentration dependence is considered. BPAF is a known endocrine disruptor that received a harmonized classification (CLH) as a reproductive toxicant category 1B under the EU CLP Regulation in 2021 and was most recently identified by the European Chemicals Agency (ECHA) as a substance of very high concern (SVHC).^39^ For the MMP assay the top 3 chemicals were the same for both REPs, but the order was BPP>BPPH> BPG based on REP_nom_ and BPPH>BPP>BPG based on REP_free_.

Based on the calculated REP_nom_ and REP_free_ of the BPA alternatives some general principles for improved comparative hazard assessment can be derived. REPs calculated from nominal effect concentrations will only provide accurate hazard ranking for stable compounds that show similar binding to medium proteins and lipids compared to the reference chemical. Based on REP_nom_, the potency of hydrophobic and highly protein-bound chemicals is underestimated if the reference chemical is more hydrophilic. This was the case for many of the BPA alternatives of the present study. Likewise, the potency of hydrophilic chemicals will be overestimated based on REP_nom_, if a reference chemical is used that is strongly bound to proteins and/or lipids.

The same concept as applied in the present study can also be used for other classes of chemicals. Abiotic stability should always be measured first. For unstable chemicals hazard assessment will need to include the formed transformation products as well and a concentration metric that considers temporal changes (e.g., area under the curve). The same applies to stable chemicals if cell lines with high metabolic capacity are used for *in vitro* testing (e.g., HepG2 or HepaRG). For stable and not quickly metabolized chemicals EC_free_ should be used for hazard ranking, if the chemicals show significant binding to medium proteins and lipids or high chemical diversity like the BPA alternatives investigated in the present study. Simple prediction models can provide a fast alternative to experimental determination of *f*_free_, but they rely on the quality of the used chemical input parameters and are less accurate for organic acids that show nonlinear protein binding.

Finally, for IVIVE, metabolism of the BPA alternatives needs to be considered as well. Metabolization does not only differ between BPA and its alternatives, but also between different cell lines and between *in vitro* and *in vivo*. For mechanistic understanding of metabolism and its influence on toxicity it might be advisible to use a defined external metabolic system as used by Srebny et al.^9^ with non-metabolically competent cell lines before testing directly in metabolically competent cells. Metabolization generally decreases estrogenicity and cytotoxicity of BPA and its alternatives,^9^ but data for other end points are largely missing.

## Supplementary Material





## Notes

Detailed protocol for SPME method optimization, figures of pin uptake kinetics in medium and PBS, pin sorption isotherms in PBS, BSA sorption isotherms, and measured freely dissolved concentrations in medium (PDF)
Tables containing more detailed information on the test chemicals, instrumental analysis, SPME parameters, measured BSA-water and membrane-water distribution ratios, and free fractions in medium (XLSX)
