# Supplementary material for: In Vitro Exposure Assessment Improves Hazard Ranking of Bisphenol A Alternatives
Source: Chem Res Toxicol. 2026 Sep 30;39(10):2099–108. doi: 10.1021/acs.chemrestox.6c00300 (PMC13685489; doi:10.1021/acs.chemrestox.6c00300)
Supplement: Supplementary file 1 [file tx6c00300_si_001.pdf]

Supporting Information
In Vitro Exposure Assessment Improves Hazard Ranking of Bisphenol A Alternatives

Luise Henneberger[†,], Sophia Bardehly[†], Arina Kremser[†], Vanessa Srebny[†], Beate I. Escher[†, §,#]*

[†] Helmholtz Centre for Environmental Research – UFZ, Department of Cell Toxicology, Permoserstr. 15, 04318 Leipzig, Germany

[§] Eberhard Karls University Tübingen, Environmental Toxicology, Department of Geosciences, 72076 Tübingen, Germany

[#]German Center for Child and Adolescent Health (DZKJ), partner site Leipzig/Dresden, Leipzig 04103, Germany

* Corresponding author

Corresponding Author Contact Info:
Luise Henneberger, Department of Cell Toxicology, Helmholtz Centre for Environmental Research − UFZ, Permoserstr. 15, 04318 Leipzig, Germany, luise.henneberger@ufz.de

Table of Contents

S1. Optimization of solid-phase microextraction (SPME) method 2

Figure S1. Pin uptake kinetics in medium. 4

Figure S2. Pin uptake kinetics in PBS. 5

Figure S3. Sorption isotherms of all test chemicals in PBS. The solid lines indicate the fitted Freundlich model, the dotted lines show a fit with a fixed slope of 1. 6

Figure S4. Pin-water distribution ($D_{pin/w}$ (pH 7.4)) and equilibration time ($t_{95\%}$) in PBS vs. membrane-water distribution ratio ($D_{m/w}$ (pH 7.4). 7

S2. Bovine serum albumin binding 8

Figure S5. BSA sorption isotherms of all test chemicals. The solid lines indicate the fitted isotherms, the dotted lines show a fit with a fixed slope of 1. 8

S3. Measured freely dissolved concentrations 9

Figure S6. Measured freely dissolved concentrations in bioassay medium normalized by the nominal concentration (C_{free}/C_{nom}). 9

References 10

S1. Optimization of solid-phase microextraction (SPME) method

Protocol

The Optimization of the SPME method included the determination of pin-water distribution ratios ($D_{pin/w}$), sorption kinetics in medium (Fig. S1) and PBS (Fig. S2) and isotherms (Fig. S3). Sorption kinetics in PBS and medium for each chemical, including equilibration time between the aqueous phase and the pin coating, were measured with the 96-pin SPME workflow. The SPME workflow was repeated for six different extraction times (15 min, 30 min, 1h, 2 h, 4 h and 24 h). The desorption time was always 2 h. The samples were extracted in triplicates and measured with instrumental analysis. The final test concentration was 1 mg/L for all chemicals except BPA (5 mg/L), BPF (5 mg/L) and BPZ (10 mg/L). To ensure reliable quantification, test concentrations were set 100- to 1000-times above the limit of quantification (LOQ). To ensure complete dissolution of each test chemicals the samples were prepared using two separate approaches: (i) preparation one day before the experiment with overnight incubation at 37°C and 250 rpm to allow complete dissolution of the compounds, and (ii) preparation directly before SPME extraction. The approach yielding the higher extracted chemical amount was used for the further experiments.

To assess the binding behavior of the selected chemicals to the pin coating, sorption isotherm experiments were performed in PBS. The test concentrations varied by 1 to 3 orders of magnitude and were based on the nominal concentration range of the *in vitro* bioassays performed by Srebny et al.[1] Three to six different concentrations were tested for each chemical. Samples were extracted in duplicates at equilibrium with the SPME pin workflow as described above with an extraction time based on the results from the sorption kinetic experiments in PBS (Table S5) and a desorption time of 2 h.

The experiments with the different extraction times, as well as the pin sorption isotherm experiments, were conducted on different days, the resulting extracts represent a combination of independent and technical replicates.

Data evaluation

The concentration of the test chemicals in the pin coating (C_{pin}) was derived from the measured concentration in the desorption solution (C_{des}), the desorption volume (V_{des}) and the coating volume (V_{pin}), as described in Eq. S1.

$$C_{pin} = \frac{C_{des} \times V_{des}}{V_{pin}} \qquad \text{Eq. S1}$$

The kinetics of chemical uptake to the pin coating were fitted using Eq. S2. $n_{pin}(t)$ represents the change of the chemical amount in the pin coating over time. $n_{pin}(eq)$ is the amount of the chemical in the pin coating at equilibrium and k_1 is the corresponding uptake rate constant.

$$n_{pin}(t) = n_{pin}(eq) \times (1-e^{-k_1 \cdot t}) \qquad \text{Eq. S2}$$

The time to reach 95% equilibrium ($t_{95\%}$) between the water phase and the pin coating was calculated using Eq. S3.

$$t_{95\%} = \frac{\ln 0.05}{-k_1} \qquad \text{Eq. S3}$$

Sorption isotherms were fitted using the log-transformed Freundlich model (Eq. S4). Freundlich coefficient and Freundlich exponent are indicated by K_{Fr} and n_{Fr}.

$$\log C_{pin} = n_{FR} \times \log C_{free} + \log K_{Fr} \qquad \text{Eq. S4}$$

The experimental sorption data were used to assess whether the pin-water distribution ratio $D_{pin/w}$ was concentration-dependent, indicated by a non-linear sorption of the chemical. Non-linear sorption was evaluated using an extra-sum of squares F-test. If n_{Fr} significantly differed from 1, it could be assumed that $D_{pin/w}$ is dependent on the test concentration. For chemicals with binding independent of concentration, pin-water distribution ratios were calculated from C_{pin} and the corresponding measured concentration in the aqueous (PBS) phase (C_w) by Eq. S5.

$$D_{pin/w} = \frac{C_{pin}}{C_w} \qquad \text{Eq. S5}$$

Medium

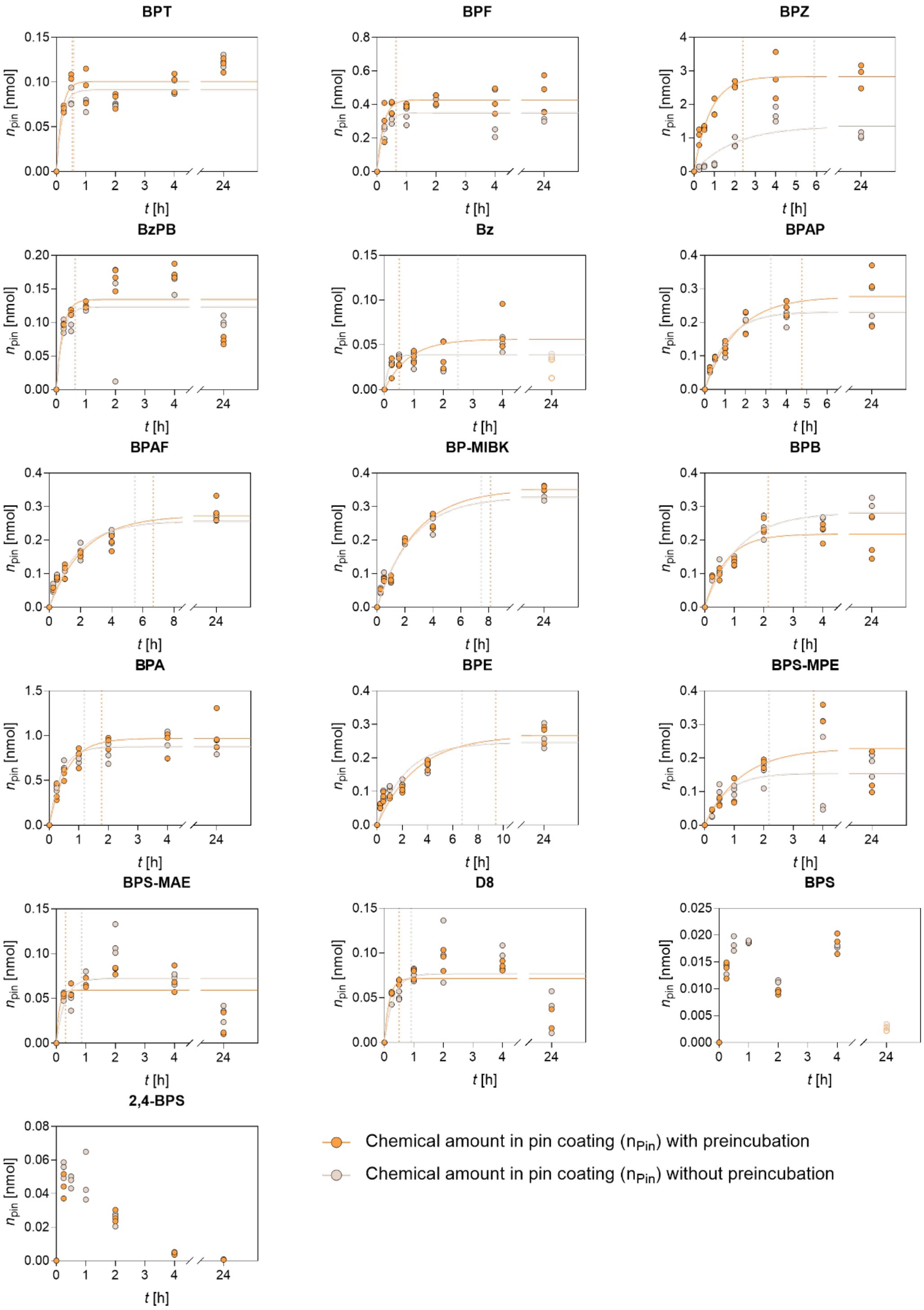

Figure S1. Pin uptake kinetics in medium.

PBS

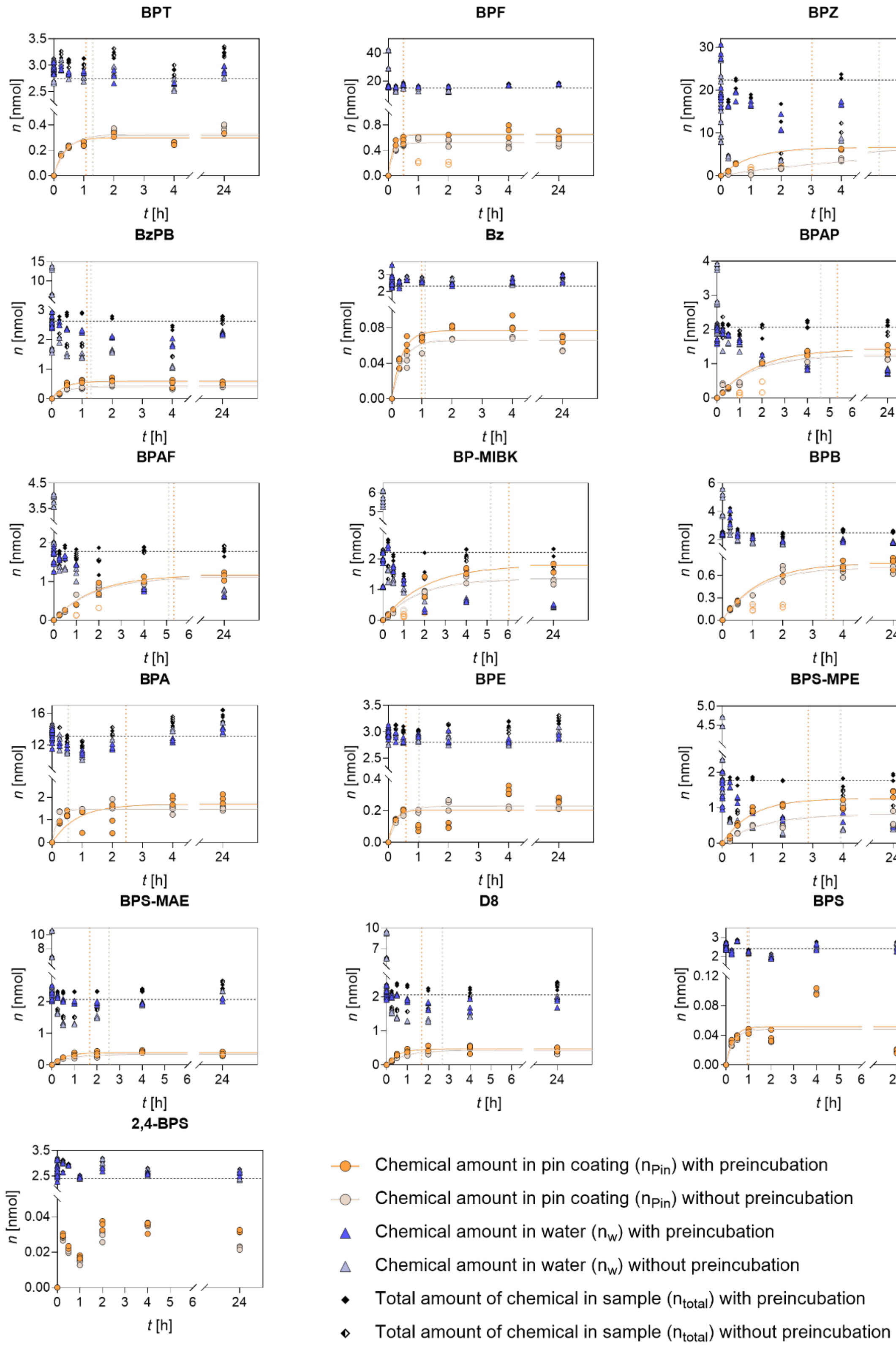

Figure S2. Pin uptake kinetics in PBS.

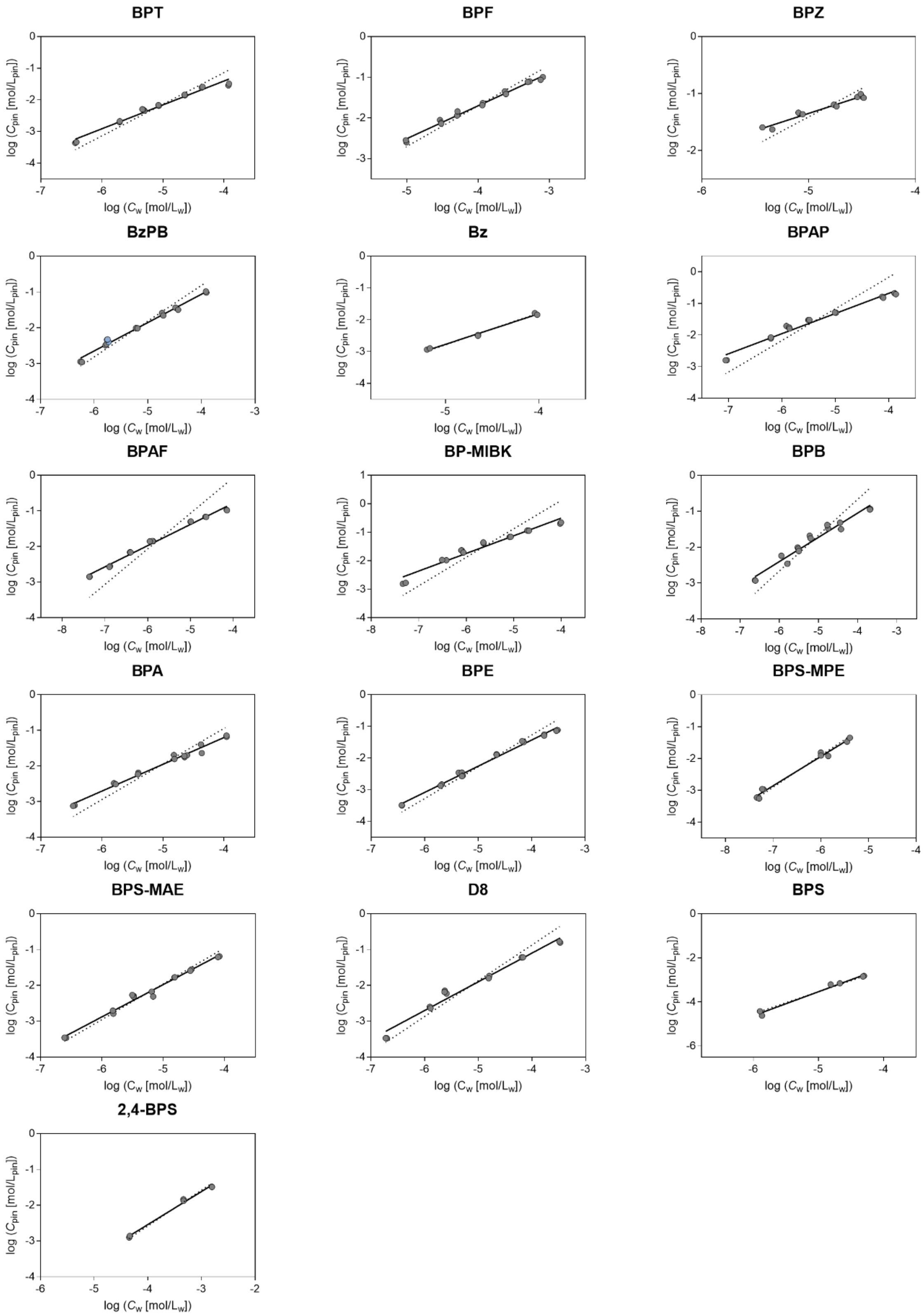

Figure S3. Sorption isotherms of all test chemicals in PBS. The solid lines indicate the fitted Freundlich model, the dotted lines show a fit with a fixed slope of 1.

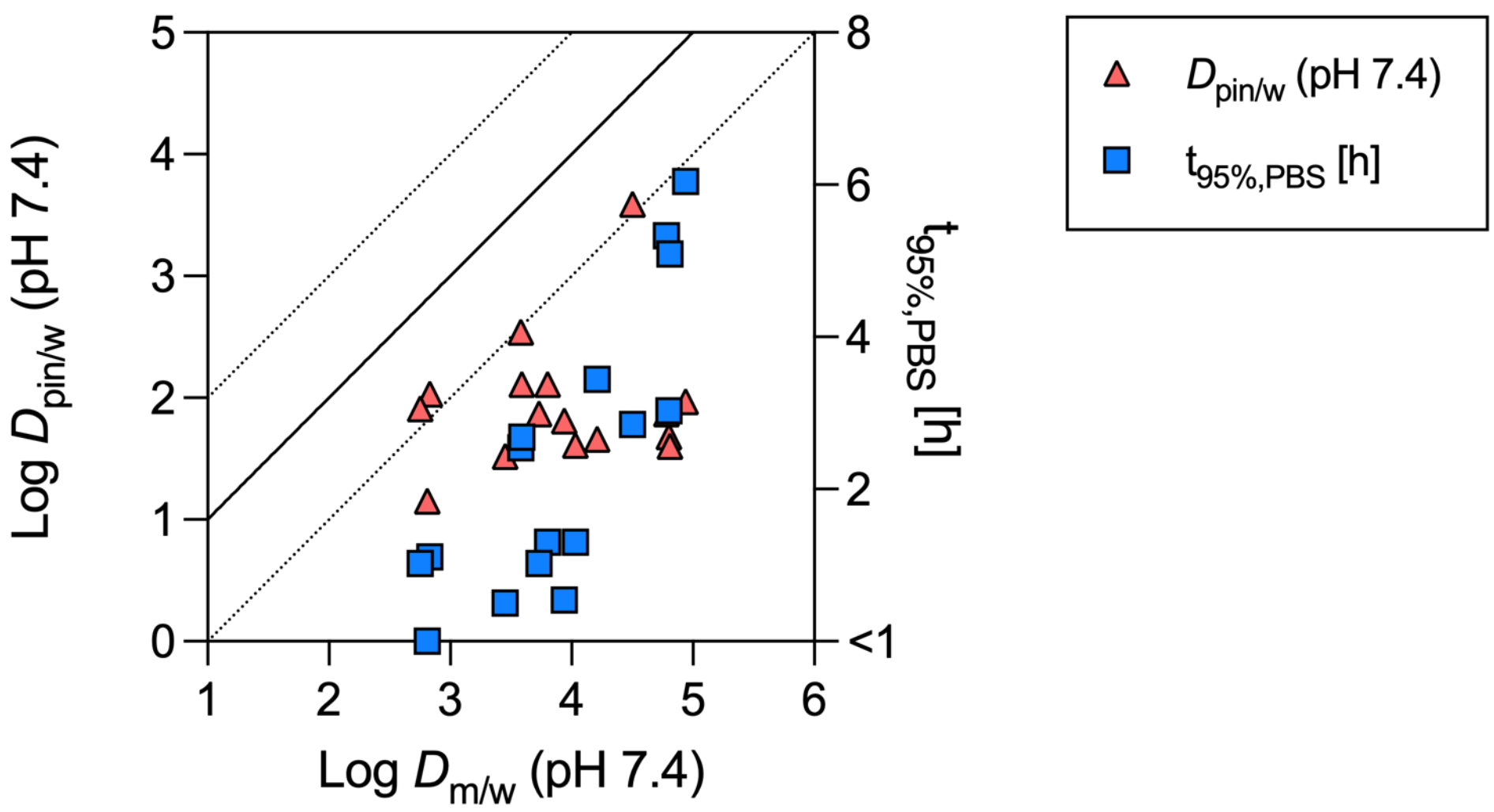

Figure S4. Pin-water distribution ($D_{pin/w}$ (pH 7.4)) and equilibration time ($t_{95\%}$) in PBS vs. membrane-water distribution ratio ($D_{m/w}$ (pH 7.4).

S2. Bovine serum albumin binding

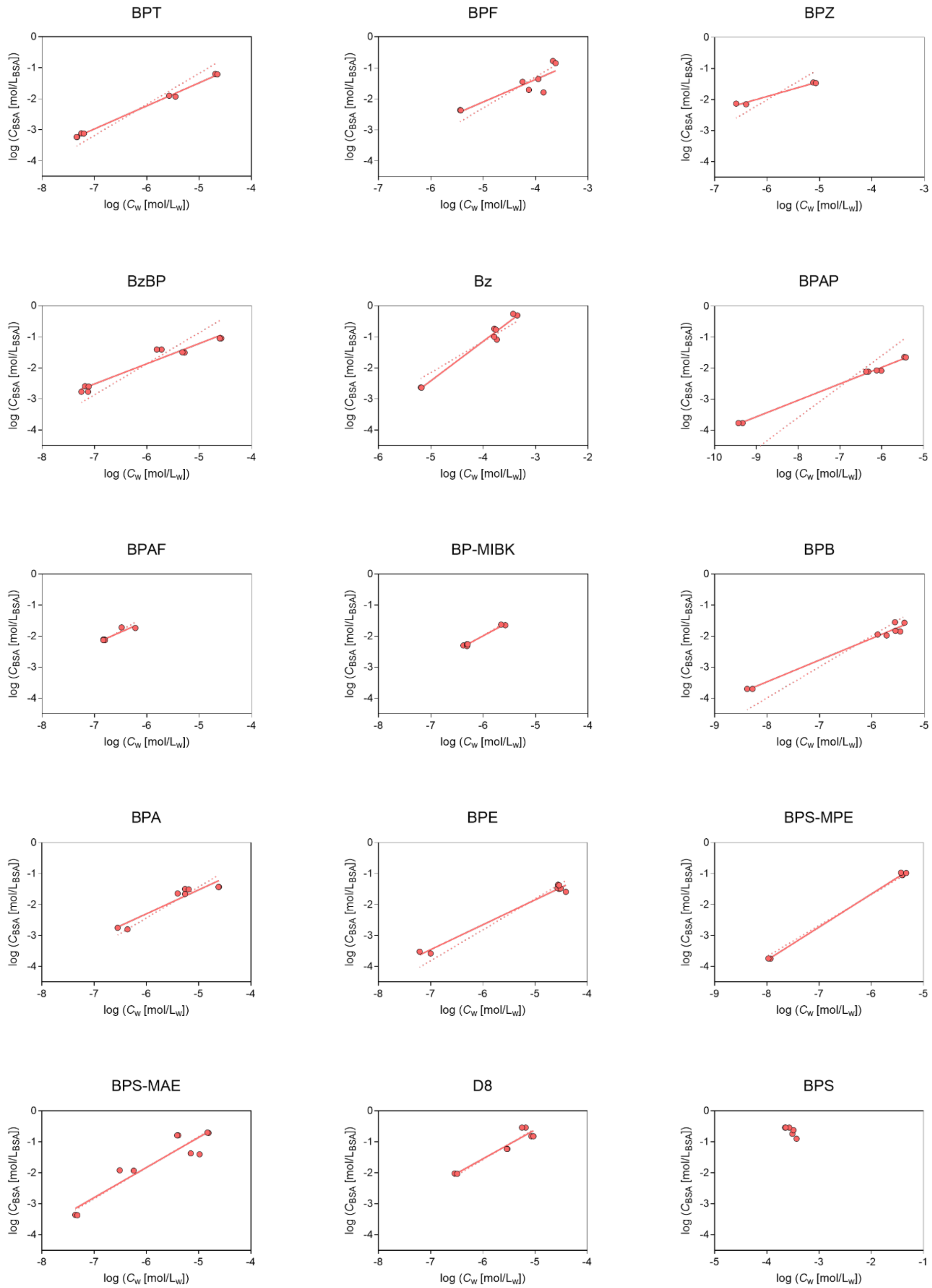

Figure S5. BSA sorption isotherms of all test chemicals. The solid lines indicate the fitted isotherms, the dotted lines show a fit with a fixed slope of 1.

S3. Measured freely dissolved concentrations

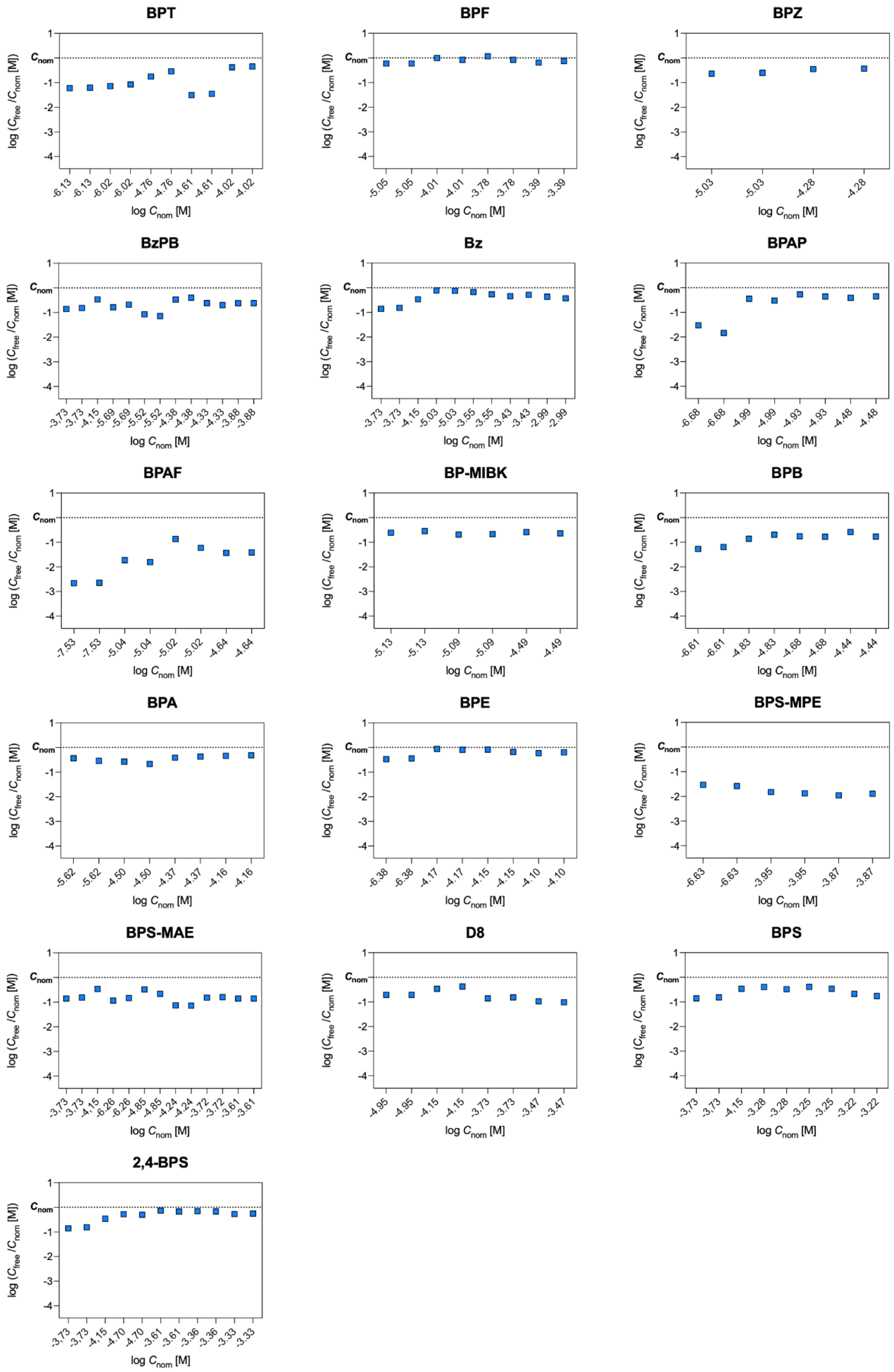

Figure S6. Measured freely dissolved concentrations in bioassay medium normalized by the nominal concentration (C_{free}/C_{nom}).

References

(1) Srebny, V.; Henneberger, L.; König, M.; Huchthausen, J.; Braasch, J.; Escher, B. I. Beyond Estrogenicity: A Comparative Assessment of Bisphenol A and Its Alternatives in In Vitro Assays Questions Safety of Replacements. *Environmental Science & Technology* **2025**. DOI: 10.1021/acs.est.5c07018.
